# Tea Polyphenols Prevent Sepsis-Induced Lung Injury via Promoting Translocation of DJ-1 to Mitochondria

**DOI:** 10.3389/fcell.2021.622507

**Published:** 2021-04-26

**Authors:** Chun-Mei Jia, Feng-Wei Zhang, Shu-Juan Wang, Wei Wang, Yong Li

**Affiliations:** Emergency Department, Cangzhou Central Hospital, Cangzhou, China

**Keywords:** sepsis, tea polyphenols, mitochondrial function, DJ-1, cecal ligation and puncture

## Abstract

**Background:**

Sepsis is the systemic inflammatory response syndrome caused by infection, which commonly targets on the lung. Tea polyphenols (TP) have many pharmacological activities, but their role in sepsis induced lung injury remains unclear.

**Results:**

Injection of TP after cecal ligation and puncture (CLP) operation elevated the survival rate in a concentration dependent manner. TP treatment improved alveoli structure injury under CLP operation. CLP surgery increased the expression of inflammatory factors IL1β, IL6, and TNFα expression, which was reversed by TP injection. In addition, CLP operation promoted apoptosis and senescence in tissues and cells during lung injury, while TP administration removed the damaged role of CLP on lung tissues and cells. Furthermore, CLP operation or LPS (lipopolysaccharide) treatment induced dysfunction of mitochondria in lung tissues and cells, but TP contributed to recover mitochondria function, which exhibited as inhibition of ROS production inhibition and increase of ATP content and Mitochondrial membrane potential (MMP). Interestingly, DJ-1 was inhibited by CLP operation but promoted by TP treatment. Overexpression of DJ-1 reversed the injury of LPS on L2 cells and recovered mitochondria normal function. And silencing of DJ-1 in rats or alveolar epithelial cells blocked the protection effect of TP.

**Conclusion:**

Our research revealed that TP protected against lung injury via upregulating of DJ-1 to improve mitochondria function, which contributed to the prevention and treatment of sepsis induced lung injury.

## Introduction

Sepsis is the systemic inflammatory response syndrome caused by infection. It often occurs in patients with severe burns, multiple injuries, and clinically critically ill patients after surgery (ARISE Investigators, ANZICS Clinical Trials Group, Peake et al., 2014). Sepsis progresses fast, accompanied by multiple organ dysfunction, and organ failure is the direct cause of death from sepsis. Especially, lung is the most vulnerable target organ when sepsis occurs (Park et al., 2019). Among the multiple organ injuries with sepsis, acute lung injury occurs earliest and has the highest incidence, and it is also one of the important causes of patient death. The mortality rate of acute lung injury caused by sepsis can reach as high as 70% if not treated in time, which is one of the critical problems facing medicine today (Proudfoot et al., 2011; Zhao et al., 2016). Looking for effective treatment strategies for acute lung injury has important clinical significance.

Tea polyphenols (TP) are the general name of polyhydroxyphenols in tea (Li et al., 2019). The main components are catechins, flavonoids, flavonols, anthocyanins, phenolic acids, phenolic acids, and polyphenols (Tamilmani and Pandey, 2016). TP have many pharmacological activities, such as anticancer, antiviral, bacteriostatic, hypoglycemic, hypolipidemic, anti-aging, and relieving acute respiratory distress syndrome (ARDS) (Wheeler et al., 2007; Reitzer et al., 2018). These effects are closely related to its strong antioxidant function. Studies on cerebral ischemia/reperfusion injury have shown that the antioxidant effect of TP can antagonize apoptosis induced by brain ischemia/reperfusion injury (Xue et al., 2016), thus improving neurological function (Lv et al., 2013). However, the function of TP in sepsis induced lung injury remains unclear.

Under the influence of various external factors and internal factors, excessive production of reactive oxygen species (ROS)/reactive nitrogen (RNS) is out of balance with antioxidant protection mechanism, leading to oxidative stress and tissue damage (Lavie, 2015). Oxidative stress can be involved in some diseases related to cell death, such as neurodegenerative diseases, tumors, inflammation (Liu et al., 2019). As the main place of energy metabolism in eukaryotes, mitochondria play an important role in physiological and pathological activities such as free radical production, apoptosis, and senescence. Further, mitochondria are also important target organelles for oxidative stress (Reiter et al., 2016). When cerebellar granule neurons treated with hydrogen peroxide, the expressions of Fis1, Drp1, and Mfn1 were decreased, which induced mitochondrial fusion. And, overexpression of proteins involved in mitochondrial fusion prevented mitochondrial division and cell death (Jahani-Asl et al., 2011). Therefore, improving mitochondrial function can inhibit the level of oxidative stress and antagonize a variety of diseases. Thus, we wonder whether TP protected against sepsis induced lung injury through regulating mitochondria function.

DJ-1 is the third gene found to be associated with familial Parkinson’s disease (PD) (Burbulla et al., 2019). DJ-1 protein is widely found in liver, skeletal muscle, kidney, brain, and testis. Current researches suggest that DJ-1 protein shows a role in antioxidant stress, anti-apoptosis, and autophagy (Kahle et al., 2009). The functions of DJ-1 protein are mainly realized through several mechanisms, including acting as scavenger of ROS and autophagy (Jiang et al., 2016; Urano et al., 2018). Under normal circumstances, DJ-1 protein is mainly located in the cytoplasm, and a small part of it is found in the nucleus and mitochondria. DJ-1 protein is not only distributed in the mitochondria, but also transcriptional regulation of some mitochondrial related proteins. Although DJ-1 protein is less distributed in the mitochondria, DJ-1 protein located in the mitochondria shows stronger cellular protection than DJ-1 protein located in the cytoplasm and nucleus (Junn et al., 2009). Moreover, DJ-1 was reported to contribute to acute lung injury, and protected lung function against inflammation (Amatullah et al., 2021, 2017; Shan et al., 2015). However, whether DJ-1 is involved in the process of TP protected against sepsis induced lung injury remains to be explored.

Present study aimed to explore whether TP protects lung from sepsis induced injury, and further seek for the underlying mechanisms, which may provide new ideas for the clinical treatment of lung injury.

## Materials and Methods

### Animal Experiment

The animal experiments were finished depended on the protocols. Female rats were provided by Cyagen Biosciences (Suzhou, China) and divided into these groups randomly (*n* = 15 per group): Sham (sham-operated), cecal ligation, and puncture (CLP) + saline (CLP model with saline injection), CLP + TP 5 (CLP model with 5 mg/kg TP injection), CLP + TP 10 (CLP model with 10 mg/kg TP injection), CLP + TP 15 (CLP model with 15 mg/kg TP injection), dexamethasone (positive control, 2 mg/kg). CLP model was established as previous described (Chen et al., 2014). Briefly, rats were anesthetized using chloral hydrate (400 mg/kg, intraperitoneal injection), before taking a ventral midline incision, cut hair through the abdominal wall, and disinfect with iodine volts on the skin surface. A 3 cm-long incision was made along the midline of the anterior abdomen, and the cecum was removed from the abdominal cavity. The cecum was ligated 1.5 cm from the blind end with a 4.0 silk thread, and the distal end of the cecum ligation was inserted twice with an 18-point needle. A little fecal matter was extruded from the intestine, and then the cecum was incorporated into the abdominal cavity and the abdomen was closed layer by layer. We injected TP (5, 10, and 15 mg/kg, purchased from MK bio, CAS 84650-60-2) and saline (24 ml/kg) in to rats through tail vein after CLP surgery twice a day for 3 days. Four hundred microliter lentivirus packaging siRNA of DJ-1 (1 × 10^9^ TU/ml) was injected in to rats through tail vein after CLP surgery. All rats were raised in SPF animal houses. Lung tissues were collected after 7 days of CLP surgery for next experiments. The experiments were finished depended on the protocols and accordance to the National Institutes of Health guidelines. This study was reviewed and approved by the Institutional animal care and use committee of Cangzhou Central Hospital.

### Cell Culture and Treatment

L2 cells (pulmonary epithelial cell) were purchased from the Science Cell Laboratory. Cells were cultured in RPMI 1640 supplemented with 10% fetal bovine serum and 100 μL/mL penicillin and streptomycin. Lipopolysaccharide (LPS) was added into cells at a concentration of 100 ng/mL for 6 h. TP was added into cells at a concentration of 5, 10, 15 μg/ml for 24 h after LPS treatment. Two microgram DJ-1 plasmid was transfected into L2 cells for 48 h.

### H&E Staining

The lung tissues were gathered and fixed in 4% paraformaldehyde for 24 h. Then, the fixed tissues were embedded in paraffin. Next, Paraffin slicer machine was used to cut slices (5-mm cross-sectional). H&E staining was used to evaluate pulmonary morphology. Lung sections were dewaxed with xylene and treated with ethanol at different concentrations for 5 min. Hematoxylin staining for 5 min, 5% acetic acid treatment for 1 min, and water rinse. Dye with eosin for 1 min, rinse with running water. Dehydrate in 70, 80, 90, 100% ethanol for 10 s, and xylene for 1 min. Drizzle with neutral gum and seal.

### Immunohistochemistry

Frozen sections of rat lung were fixated in 4% paraformaldehyde and washed using PBS. We penetrated sections using 0.5% Triton X-100. After three times wash, we blocked sections with 50% goat serum. Then, sections were incubated with E-cadherin antibody overnight. Then, we incubated the sections using secondary antibody. Immunofluorescence was analyzed under an IX73 fluorescence microscope (Olympus, Valley, PA).

### qRT-PCR

Total RNA was isolated from serum and culture medium according to a standard protocol. And then, the purity and concentration of RNA was detected and all the samples were converted into cDNA using reverse transcription kit. We used the SYBR Green (Thermo Fisher Scientific) system to perform the qRT-PCR. Data was analyzed by GraphPad 7 (Table 1).

**TABLE 1:** Primer sequences for reverse transcription quantitative polymerase chain reaction.

Targets	Forward	Reverse
IL1β	GAAATGCCACCTTTTGACAGTG	TGGATGCTCTCATCAGGACAG
IL6	CACAGCAAGGCCTAGGAAAG	TTGGTTCAGCCACTGCCGTA
TNFα	TGGATGCTCTCATCAGGACAG	TGGATGCTCTCATCAGGACAG
E-cadherin	CAGTTCCGAGGTCTACACCTT	TGAATCGGGAGTCTTCCGAAAA
Sftpc	CAGCTCCAGGAACCTACTGC	CACAGCAAGGCCTAGGAAAG
F VII	ATGACGACTGCCATCCTAGAG	GCTCCCTAAAGAGCTGGGG
p53	TCACAGCGTCTGTTGACATTT	ACCAAGCTCATTACCCTGACA
p21	AGAGAAAAGCCCGTACTTTCAG	GGGCAGCCTGTGATTCCAT
GAPDH	ATGTCGTGG AGTCTACTGGC	TGACCTTGCCCACAGCCTTG

### TUNEL

We used the *in situ* Cell Death Detection Kit (TUNEL fluorescence FITC kit, Roche, Germany) detect apoptotic. We used the DAPI to stain nuclei. We used the IX73 fluorescence microscope (Olympus, Valley, PA) to analyze fluorescence staining. We used the Image-J to count the Total cells and TUNEL positive cells numbers.

### Western Blot

Protein samples were blotted depended on standard protocol. And we used the Odyssey Infrared Scanning System (Gene Co., Ltd., Hongkong, China) to scan the membranes. At last, we used the Image J software to analyze the western bolt results. The antibodies are as list: Bax, Caspase-3, Caspase-9, p21, p53, DJ-1, and GAPDH antibody were produced by Proteintech Group (Wuhan, China). The secondary antibodies IRDye700/800 Mouse or Rabbit were produced by LICOR (Lincoln, Nebraska, United States).

### MTT Assay

L2 cells were plated in 96-well plates and we used MTT assay to detect the cell viability. MTT (0.5 mg/mL; Beyotime Biotechnology, China) was added to every well after treatment and incubated for 3 h at 37°C. And, 150 μL DMSO was added and incubated for 15 min. We measured the absorbance by the Spectrophotometer (Tecan, Austria) at 493 nm.

### ROS Assay

Reactive oxygen species detection was performed according to the procedures (Beyotime, China). Briefly, L2 cells were plated in 12-well plates and ROS solution was added into cells for 20 min. After fixation in 4% paraformaldehyde and PBS washing solution, the cells were incubated in DAPI for 10 min. Fluorescence was observed by the fluorescence microscope.

### ATP Content Detection

ATP was determined by the ATP Assay Kit (Beyotime, China). L2 cells were plated in 12-well plates and added lysate solution. The supernatant was centrifuged at 4°C at 12,000 × g for 5 min. ATP detection buffer was added and analyzed by fluorescence spectrometry. The final ATP content of each sample was normalized to its protein concentration with the BCA protein detection kit (Beyotime, China).

### Statistical Analysis

All measurement data were shown as mean ± SD and analyzed by the Graphpad 7.0 and SPSS 22.0 software, with *p* < 0.05 as a level of statistically significance. Data between two groups were compared by unpaired *t*-test, while those among multiple groups were tested using one-way analysis of variance (ANOVA), followed by the Tukey’s *post hoc* test. Comparison of data at different time points was analyzed using two-way ANOVA. Data were examined by the Shapiro-Wilk normality test.

## Results

### TP Increased Survival Rate and Improved Pulmonary Epithelial Function in the Lung Tissues of Septic Rats

**FIGURE 1:**
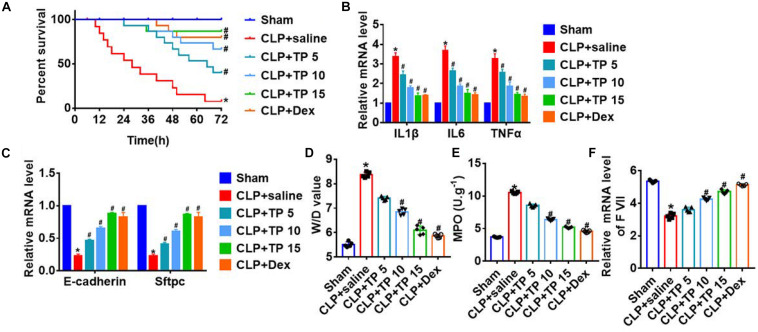
CLP increased survival and improved pulmonary epithelial function in response to CLP operation. Rats were suffered from CLP or sham surgery with or without TP (5, 10, and 15 mg/kg) and saline (24 ml/kg): Sham (*n* = 15), CLP (CLP model with saline injection, *n* = 15), CLP + TP 5 (CLP model with 5 mg/kg TP injection, *n* = 15), CLP + TP 10 (CLP model with 10 mg/kg TP injection, *n* = 15), CLP + TP 15 (CLP model with 15 mg/kg TP injection, *n* = 15). And, dexamethasone (2 mg/kg) served as a positive control. **(A)** Survival plots for rats in different groups. *P < 0.05 vs. Sham, ^#^*P* < 0.05 vs. CLP. **(B)** H&E staining for lung sections. **(C)** The mRNA expression of inflammatory factors (IL1β, IL6, and TNFα). *n* = 4; *P < 0.05 vs. Sham, ^#^P < 0.05 vs. CLP. **(D)** Quantitative RT-PCR was performed to analyze the mRNA expression of E-cadherin (Cdh1) and Sftpc. *n* = 5, *P < 0.05 vs. Sham, ^#^*P* < 0.05 vs. CLP. **(E)** Wet dry mass ratio (W/D) of lungs was calculated. *n* = 5, *P < 0.05 vs. Sham, ^#^*P* < 0.05 vs. CLP. **(F)** Myeloperoxidase (MPO) of lungs was examined. *n* = 5, *P < 0.05 vs. Sham, ^#^*P* < 0.05 vs. CLP. The mRNA level of F VII was detected using qRT-PCR. *n* = 5, *P < 0.05 vs. Sham, ^#^*P* < 0.05 vs. CLP.

To explore the function of TP in sepsis induced lung injury, rats were suffered from CLP or sham surgery with or without TP (5, 10, and 15 mg/kg) and saline (24 ml/kg) or dexamethasone (2 mg/kg). After 72 h of surgery, the survival rate of CLP + saline group almost decreased to 0%, while injection of TP after CLP operation elevated the survival rate in a concentration dependent manner (Figure 1A). And, the H&E staining showed a normal alveoli structure, while CLP operation induced a significantly vascular congestion and pulmonary edema. TP treatment improved alveoli structure injury under CLP operation (Supplementary Figure 1). Inflammation is common during lung injury, and we found CLP surgery increased the expression of inflammatory factors IL1β, IL6, and TNFα expression, which was reversed by TP injection (Figure 1B). Alveolar epithelial cells are involved in maintaining normal lung function, so we estimated alveolar epithelial cells function by examining epithelial markers expression. As well, the mRNA expressions of E-cadherin and Sftpc (Surfactant C protein) were reduced in CLP + saline group compared with sham group. Nevertheless, TP treatment increased the expression of E-cadherin and Sftpc in a concentration dependent manner (Figure 1C). CLP surgery increased wet dry mass ratio (W/D) of isolated lungs, while TP treatment decreased W/D, indicating that TP treatment promotes pulmonary edema (Figure 1D). As well, TP treatment inhibited the increase of myeloperoxidase (MPO) in CLP group (Figure 1E). And, TP treatment induced the expression of coagulation factor VII (F VII) that was reduced upon CLP surgery (Figure 1F).

These findings suggested that TP treatment played a protective role in lung injury induced by sepsis.

### TP Attenuated CLP and LPS-Induced Apoptosis in Lung Tissues and Cells

**FIGURE 2:**
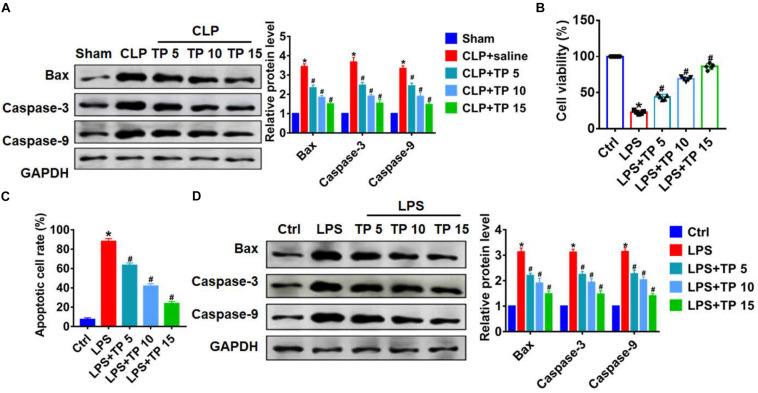
TP attenuated apoptosis during lung injury. **(A)** Western blot for apoptosis related proteins (Bax, Caspase-3, Caspase-9) in lung tissues. *n* = 5, *P < 0.05 vs. Sham, ^#^*P* < 0.05 vs. CLP. L2 cells treated with LPS (100 ng/mL) at different concentration of TP: 0, 5, 10, 15 μg/ml. **(B)** MTT was used to detect cell viability. *n* = 10, *P < 0.05 vs. control, ^#^*P* < 0.05 vs. LPS. **(C)** Apoptosis cell numbers were tested by TUNLE staining. *n* = 5, *P < 0.05 vs. control, ^#^*P* < 0.05 vs. LPS. **(D)** Western blot for apoptosis related proteins (Bax, Caspase-3, Caspase-9) in L2 cells. *n* = 5, *P < 0.05 vs. control, ^#^*P* < 0.05 vs. LPS.

It has been reported that apoptosis is common during lung injury (Hsu et al., 2019). In this study, we performed western blot to detect apoptosis related proteins level, and found that CLP operation significantly promoted the expression of Bax, Caspase-3, and Caspase-9, indicating an increase of apoptosis (Figure 2A). Whereas, TP injection inhibited Bax, Caspase-3, and Caspase-9 expression in lung tissues suffered from CLP surgery (Figure 2A). *In vitro*, we cultured L2 cells treated with LPS (100 ng/mL) to mimic *in vivo* septic alveolar epithelium. MTT results showed that LPS treatment decreased cell viability, while TP administration recover cell viability and remitted the benefit effects of LPS. Interestingly, the function of TP was concentration-dependent (Figure 2B). In addition, TUNEL analysis exhibited an increase of apoptotic cell numbers in LPS treated L2 cells, while TP administration decreased apoptosis in L2 cells (Figure 2C and Supplementary Figure 2). As well, LPS promoted the expression of Bax, Caspase-3, and Caspase-9, which was reversed by TP at different concentrations (Figure 2D). Together, TP treatment suppressed apoptosis in sepsis induced lung injury.

### TP Inhibited Senescence in Lung Tissues and Cells

**FIGURE 3:**
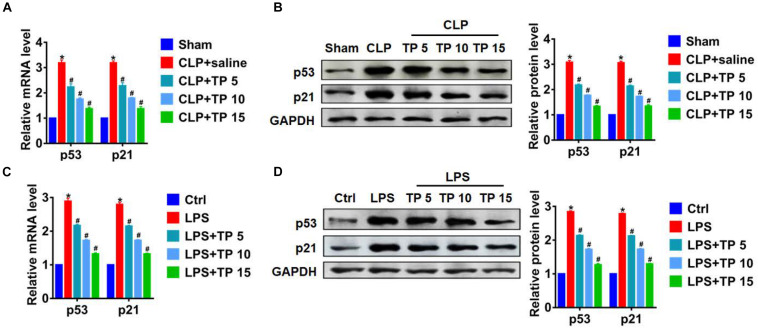
TP inhibited senescence during lung injury. **(A)** qRT-PCR of the mRNA expression of p53 and p21 in lungs from rats. *n* = 5, *P < 0.05 vs. Sham, ^#^*P* < 0.05 vs. CLP. **(B)** Western blot analysis of p53 and p21 protein expression in lungs from rats. *n* = 5, *P < 0.05 vs. Sham, ^#^*P* < 0.05 vs. CLP. **(C)** qRT-PCR for mRNA expression of p53 and p21 in L2 cells. *n* = 5, *P < 0.05 vs. control, ^#^*P* < 0.05 vs. LPS. **(D)** Western blot analysis for p53 and p21 protein expression in L2 cells. *n* = 5, *P < 0.05 vs. control, ^#^*P* < 0.05 vs. LPS.

Numerous studies have shown that alveolar epithelial senescence is involved in the process of lung injury (Borok et al., 2020). Herein, we examined the expression of senescence markers p53 and p21. qRT-PCR and western blot analysis showed CLP operation promoted senescence of lung tissues, while TP treatment inhibited p53 and p21 expression in a concentration dependent manner (Figures 3A,B). What’s more, LPS also induced an increase of p53 and p21 in L2 cells, while TP treatment decreased p53 and p21 expression (Figures 3C,D). These data suggested that TP suppressed CLP and LPS induced alveolar epithelial cell senescence.

### TP Reversed CLP and LPS-Induced Mitochondrial Abnormalities in Lung Injury

**FIGURE 4:**
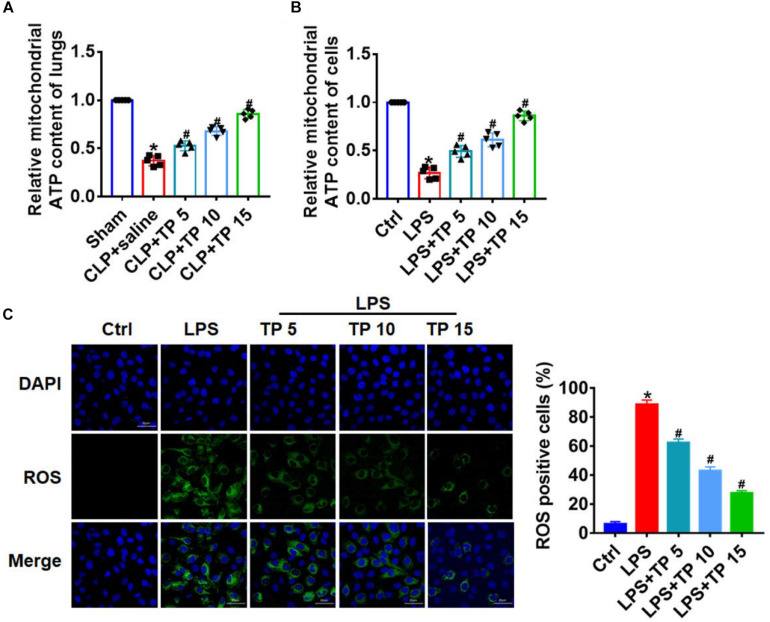
TP reversed CLP/LPS-induced mitochondrial abnormalities. **(A)** ATP content was detected in lungs from rats. *n* = 5, *P < 0.05 vs. Sham, ^#^*P* < 0.05 vs. CLP. **(B)** ATP content for L2 cells. *n* = 10, *P < 0.05 vs. control, ^#^*P* < 0.05 vs. LPS. **(C)** ROS assay for L2 cells. *n* = 5, *P < 0.05 vs. control, ^#^*P* < 0.05 vs. LPS.

Considering that mitochondria are important organelles that mediate apoptosis and senescence, we then evaluated the role of TP on mitochondrial function. ATP content can reflect the function of mitochondria, so we detected ATP content in CLP induced lung injury. CLP surgery significantly decreased ATP content in lung tissues, while TP injection removed this damage effect of CLP (Figure 4A). Then, we performed following experiments in L2 cells treated with LPS. Interestingly, LPS treatment decreased ATP content, while TP recovered ATP content in L2 cells (Figure 4B). Oxidative stress is an important link in mitochondrial initiation of apoptosis and senescence. We then examined ROS level in L2 cells treated with LPS. ROS assay showed that LPS treatment promoted ROS production, while TP inhibited ROS level in L2 cells (Figure 4C). These data indicated that TP promoted mitochondria function in sepsis induced lung injury.

### DJ-1 Participated in the Process of by Which TP Prevents Lung Injury

**FIGURE 5:**
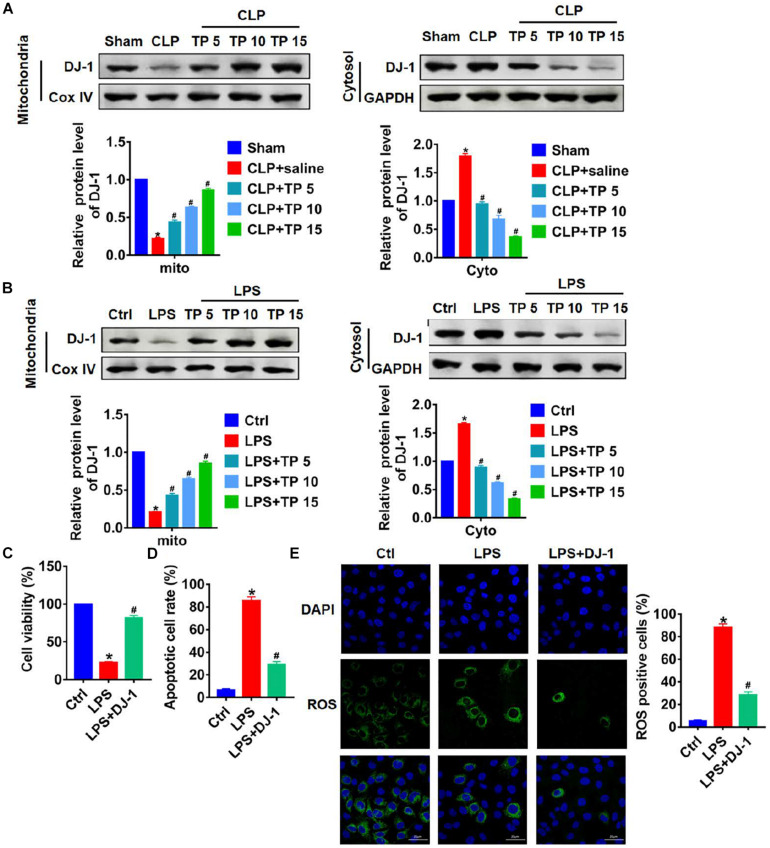
TP prevented lung injury through modulating translocation of DJ-1 from cytoplasm to mitochondria. **(A)** Western blot analysis for DJ-1 protein expression of mitochondria and cytosol in lungs from rats. *n* = 5, *P < 0.05 vs. Sham, ^#^*P* < 0.05 vs. CLP. **(B)** Western blot analysis for DJ-1 protein expression of mitochondria and cytosol in L2 cells. *n* = 5, *P < 0.05 vs. control, ^#^*P* < 0.05 vs. LPS. L2 cells were transfected with DJ-1 or NC and treated with LPS. **(C)** MTT for cell viability. *n* = 5, *P < 0.05 vs. control, ^#^*P* < 0.05 vs. LPS. **(D)** Apoptosis cell numbers were tested by TUNLE staining. *n* = 5, *P < 0.05 vs. control, ^#^*P* < 0.05 vs. LPS. **(E)** ROS assay for L2 cells. *n* = 5, *P < 0.05 vs. control, ^#^*P* < 0.05 vs. LPS.

It has reported that DJ-1 plays an important role in maintaining mitochondria function and fighting oxidative stress (Salazar et al., 2018). In this study, we speculate that TP might play a protective role by regulating DJ-1 in sepsis induced lung injury. Proteins from cytoplasm and mitochondria were extracted in CLP or LPS damaged lung tissues or cells. As shown in Figure 5A, the expression of DJ-1 was decreased in mitochondria, which was reversed upon TP treatment. On the contrary, CLP induced accumulation of DJ-1 in cytoplasm, and TP treatment decreased mitochondrial DJ-1 level. This change in the expression of DJ-1 in cytoplasm and mitochondria indicated CLP blocked the translocation of DJ-1 from cytoplasm to mitochondria, but TP recovered translocation ability of DJ-1. Consistent with dada from *in vivo* experiments, LPS treatment blocked the translocation of DJ-1 from cytoplasm to mitochondria in L2 cells, which was remitted by TP administration (Figure 5B). To further prove the role of DJ-1 in lung injury, L2 cells were transfected with DJ-1 or NC and treated with LPS at the same time. MTT results showed that the inhibitory effect of LPS on cell viability was removed by DJ-1 but not NC (Figure 5C). As well, DJ-1 inhibited LPS induced apoptosis and ROS production (Figures 5D,E and Supplementary Figures 3). Taken together, DJ-1 was involved in protection of sepsis induced lung injury.

### Silencing DJ-1 Inhibited the Effect of TP on Lung Protection

**FIGURE 6:**
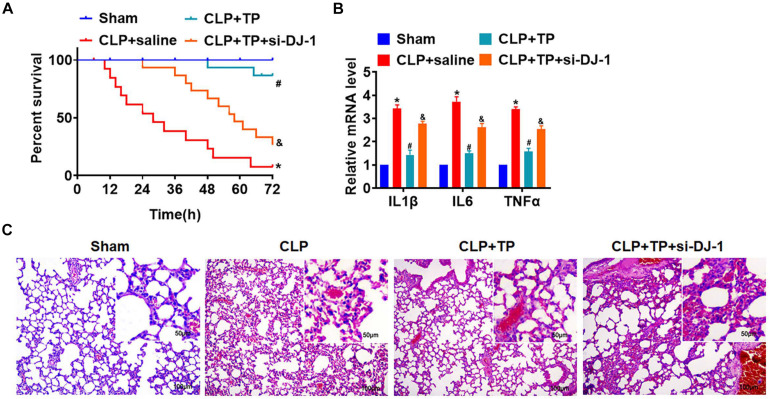
Blocking DJ-1 inhibited the effect of TP on lung injury *in vivo*. Rats were suffered from CLP or sham surgery with or without TP (15 mg/kg), and rats were injected with lentivirus containing si-DJ-1. **(A)** Survival curves of rats. **(B)** H&E staining for lung sections. **(C)** The mRNA expression of inflammatory factors (IL1β, IL6, and TNFα). *n* = 4; *P < 0.05 vs. Sham, ^#^*P* < 0.05 vs. CLP.

To identify whether TP protected lung tissues and cells from injury via regulating DJ-1, rats were suffered from CLP or sham surgery with or without TP (15 mg/kg), and rats were injected with lentivirus containing si-DJ-1. TP increased survival rate that was inhibited by CLP operation, but si-DJ-1 break the protection of TP on lung injury (Figure 6A). And, the H&E staining showed silencing of DJ-1 removed the protective effect of TP on alveoli structure (Figure 6B). TP administration inhibited the increase of inflammatory factors IL1β, IL6, and TNFα expression upon CLP in lung tissues, which was reversed by si-DJ-1 injection (Figure 6C).

**FIGURE 7:**
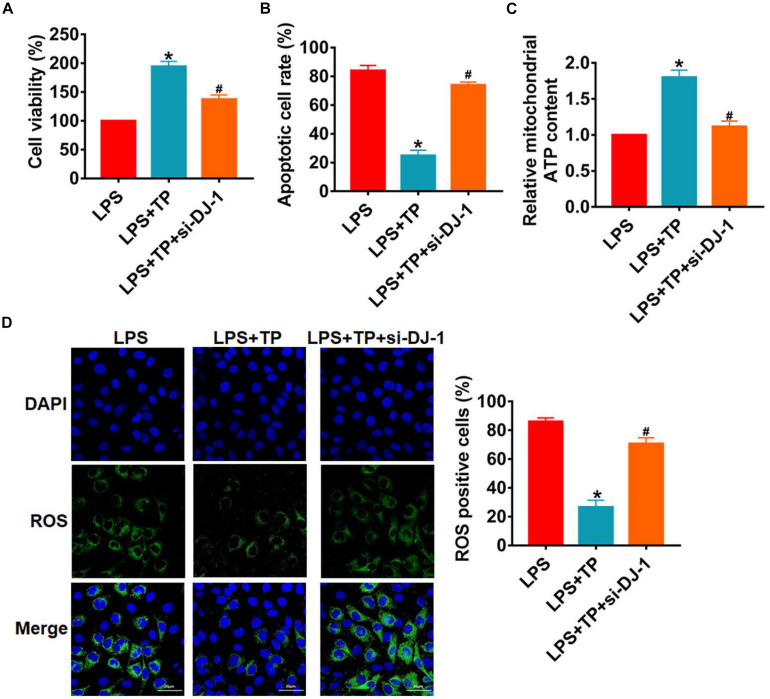
Silencing DJ-1 inhibited the effect of TP on lung cells injury *in vitro*. L2 cells were treated with LPS with or without TP (15 μg/ml) and transfected with si-DJ-1 or NC. **(A)** MTT for cell viability. *n* = 5, *P < 0.05 vs. control, ^#^*P* < 0.05 vs. LPS. **(B)** Apoptosis cell numbers were tested by TUNLE staining. *n* = 5, *P < 0.05 vs. control, ^#^*P* < 0.05 vs. LPS. **(C)** ROS assay for L2 cells. *n* = 5, *P < 0.05 vs. control, ^#^*P* < 0.05 vs. LPS. **(D)** ATP content for L2 cells. *n* = 10, *P < 0.05 vs. control, ^#^*P* < 0.05 vs. LPS.

Then, L2 cells were treated with LPS with or without TP (15 μg/ml) and transfected with si-DJ-1 or NC. MTT assay showed loss of DJ-1 inhibited cell viability and removed the protective role of TP (Figure 7A). As well, silencing of DJ-1 promoted cell apoptosis and ROS production (Figure 7B and Supplementary Figures 4 and Figure 7C). What’s more, deficiency of DJ-1 inhibited ATP content (Figure 7D), which indicated a destruction of mitochondria function. Thus, TP protected against sepsis induced lung injury by up-regulating DJ-1 expression.

## Discussion

Acute lung injury is a common clinical syndrome in heavy patients, mainly manifested as destruction of alveolar capillary barrier function and interstitial edema (Nugent et al., 2019). At present, it is believed that the pathogenesis of sepsis induced lung injury is mainly the inflammatory response, and the activation of inflammatory cells and the release of inflammatory factors induce the apoptosis and oxidative stress injury of alveolar epithelial cells, and eventually leads to alveolar epithelial dysfunction (Chen et al., 2020). Therefore, fighting against inflammation, apoptosis, and oxidative stress is an important means to treat sepsis induced lung injury, which is of great significance for restoring homeostasis and improving organ failure.

TP is not only an effective ROS scavenger for biological organisms, but also acts as a strong reducing agent for redox reaction with biological organisms to remove oxygen radicals and lipid free radicals, which can prevent lipid peroxidation, protect DNA molecules from damage, inhibit tumor development, and delay aging (Mao et al., 2019). Current studies have shown that when animals are stimulated by extracellular environment, on the one hand, TP regulates the activity of nuclear transcription factors by inhibiting phosphorylation through activation of signal transduction pathways such as nuclear transcription factor activating protein 1 (AP-1) and nuclear factor kappa B (NF-κB) (Balasubramanian et al., 2002; Rothenberg and Zhang, 2019). On the other hand, TP mainly regulates mitogen activated protein kinases (MAPK)—nuclear factor erythroid-2-related factor 2 (Nrf2)—antioxidant response element (ARE) signal transduction pathway to achieve the effect of related gene expression and protein expression affecting cell apoptosis (Owuor and Kong, 2002).

In this study, we explored the role of TP in sepsis induced lung injury. To construct sepsis model, the rats were suffered from CLP surgery. CLP surgery is a common method to induce sepsis in mice or rats (Lo et al., 2013; Xiong et al., 2020). Then, rats suffered from CLP surgery was injected with TP at three different concentrations. To further confirm the role of TP in lung injury, we detected alveolar epithelial cells markers (E-cadherin and Sftpc), wet dry mass ratio (W/D), and myeloperoxidase (MPO) and coagulation factor VII (F VII) expression of isolated lungs, these indexes are commonly used in acute lung injury detection (Xu et al., 2015). Interestingly, we found that injection of TP elevated the survival rate and improved alveoli structure injury in a concentration dependent manner. CLP surgery increased the expression of inflammatory factors IL1β, IL6, and TNFα expression, which was reversed by TP injection. In addition, CLP operation promoted apoptosis and senescence in tissues and cells during lung injury, while TP administration removed the damaged role of CLP on lung tissues and cells. Lipopolysaccharide (LPS) is the component of outer cell wall of gram-negative bacteria, which is the main pathogenic substance in sepsis-induced lung injury. And researches have shown that LPS-induced lung epithelial cell injury can be simulated *in vivo* in sepsis lung injury (Su et al., 2012; Zhang et al., 2020). Thus, to determine the protective effect of TP on alveolar epithelium, L2 cells, one kind of alveolar epithelial cell lines, were treated with 100 ng/mL LPS. Consistent with the result *in vivo*, TP attenuated apoptosis, senescence, and ROS production upon LPS treatment.

Given that mitochondria are not only organelles that provide ATP, but also important sites for apoptosis, senescence, and oxidative stress (Lin et al., 2018). We hypothesized that TP protected mitochondrial function during lung injury. As expected, CLP operation or LPS treatment induced dysfunction of mitochondria in lung tissues and cells, but TP contributed to recover mitochondria function, which exhibited as inhibition of ROS production inhibition and increase of ATP content and Mitochondrial membrane potential (MMP). Present study used three doses of TP (5, 10, and 15 mg/kg) in CLP-induced rats, and found that 15 mg/kg TP showed the highest protective effect during lung injury. Considering the extrapolation of dose between species (Nair and Jacob, 2016), the dose of TP in the clinical treatment of acute lung injury still needs to be carefully explored. Nevertheless, the differences between the animal species subjected to research and humans may also hamper the correct interpretation, extrapolation, and practical application. Besides, present study indicated that TP protected against lung injury, the main four catechins are epicatichin (EC), epigallocatichin (EGC), epicatichin gallate (ECG), and epigallocatichingallate (EGCG), among which EGCG has the highest antioxidant activity, accounting for 50% of the catechins (Salah et al., 1995). So, we think that these catechins may be the active components in TP protecting against lung injury. Thus, we will continue to pay attention on these active comments in the follow-up research.

DJ-1 is a key molecule in antioxidant defenses and maintaining mitochondria normal function, and it has been reported that translocation of DJ-1 from cytosol to mitochondria fought against hypoxia/reoxygenation-induced damage to cardiomyocytes (Deng et al., 2019), and this translocation might be mediated by Glucose regulated protein 75 (Grp75) (Zhou et al., 2020). In this study, DJ-1 was inhibited by CLP operation but promoted by TP treatment. And, overexpression of DJ-1 reversed the injury of LPS on L2 cells and recovered mitochondria normal function. But, silencing of DJ-1 in rats or alveolar epithelial cells blocked the protection effect of TP, which indicated that DJ-1 was a key molecular in TP protecting against sepsis induced lung injury. Under oxidative stress, the isoelectric point of DJ-1 protein will decrease and the acidic migration will occur. Therefore, it can be considered that DJ-1 protein is sensitive to oxidative stress of cells. Previous studies have found that DJ-1 protein can be used as a reactive oxygen scavenger and can remove hydrogen peroxide through self-oxidation *in vitro* cell models (Taira et al., 2004), thus protecting mitochondria from damage. In addition, under the stimulation of oxidative stress, DJ-1 protein can not only prevent the activation of apoptosis pathway by oxidizing its own Cys106 (Bahmed et al., 2019), but also reduce the expression of Bax protein by reducing the transcriptional activity of p53, and then inhibit the apoptotic pathway of Bax-caspases, thus protecting mitochondrial function (Salah et al., 1995). The overexpression of DJ-1 protein can protect cells, enhance the tolerance of cells to toxins, and reduce mitochondrial damage. Considering that DJ-1 protein is sensitive to oxidative stress, and TP has an antioxidant stress effect, we speculated that TP might modulated DJ-1 transposition through modulating ROS. Considering that DJ-1 protein is sensitive to oxidative stress, and TP has an antioxidant stress effect, we speculated that TP might modulated DJ-1 transposition through modulating ROS, and we will focus on this question in our future studies.

## Conclusion

In conclusion, our research revealed that TP protected against lung injury via upregulating of DJ-1 to improve mitochondria function, which contributed to the prevention and treatment of sepsis induced lung injury.

## Data Availability Statement

The original contributions presented in the study are included in the article/Supplementary Material, further inquiries can be directed to the corresponding author/s.

## Ethics Statement

The animal study was reviewed and approved by Cangzhou Central Hospital.

## Author Contributions

C-MJ and F-WZ contributed to the study design and reviewed the manuscript. F-WZ and S-JW analyzed the data and wrote the manuscript. WW and YL contributed to the data collection, data interpretation, and manuscript writing. All authors read and approved the final manuscript.

## Conflict of Interest

The authors declare that the research was conducted in the absence of any commercial or financial relationships that could be construed as a potential conflict of interest.
