# Supplementary material for: Tea Polyphenols Prevent Sepsis-Induced Lung Injury via Promoting Translocation of DJ-1 to Mitochondria
Source: Front Cell Dev Biol. 2021 Apr 26;9:622507. doi: 10.3389/fcell.2021.622507 (PMC8107366; doi:10.3389/fcell.2021.622507)
Supplement: Supplementary file 1 [file Table_1.DOCX]

**Supplementary materials**


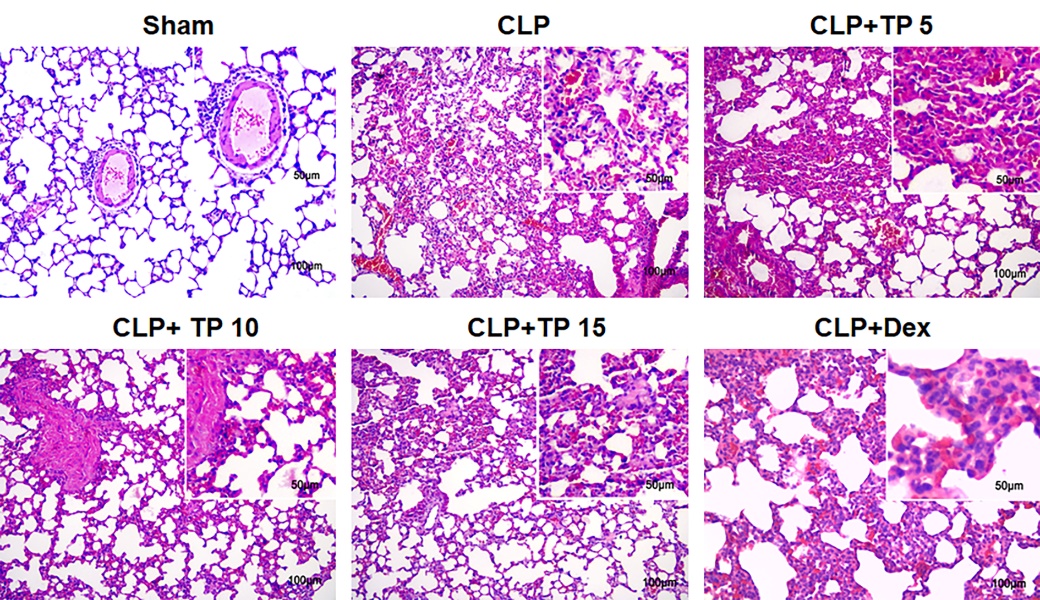
**Supplementary figure 1.** Rats were suffered from CLP or sham surgery with or without TP (5, 10 and 15 mg/kg) and saline (24ml/kg): Sham (n=15), CLP (CLP model with saline injection, n=15), CLP+ TP 5 (CLP model with 5 mg/kg TP injection, n=15), CLP+ TP 10 (CLP model with 10 mg/kg TP injection, n=15), CLP+ TP 15 (CLP model with 15 mg/kg TP injection, n=15). And dexamethasone (2 mg/kg) served as a positive control. H&E staining used to stain lung sections.

**Supplementary figure 2.** L2 cells treated with LPS (100 ng/mL) at different concentration of TP: 0, 5, 10, 15 μg/ml. Apoptosis cell numbers were tested by TUNLE staining.


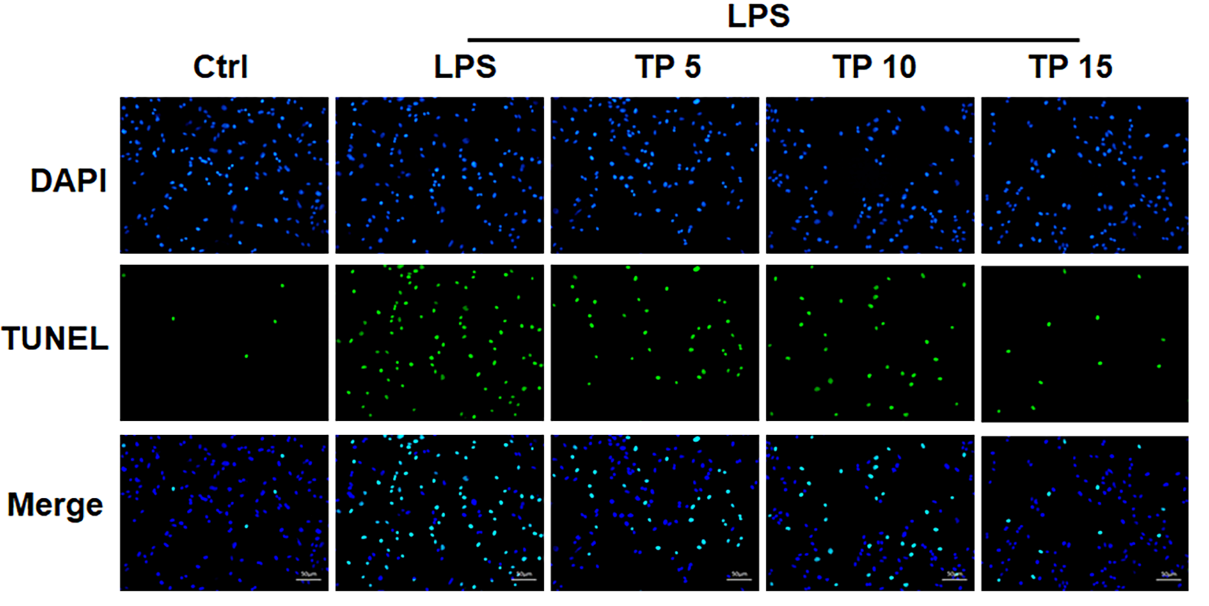


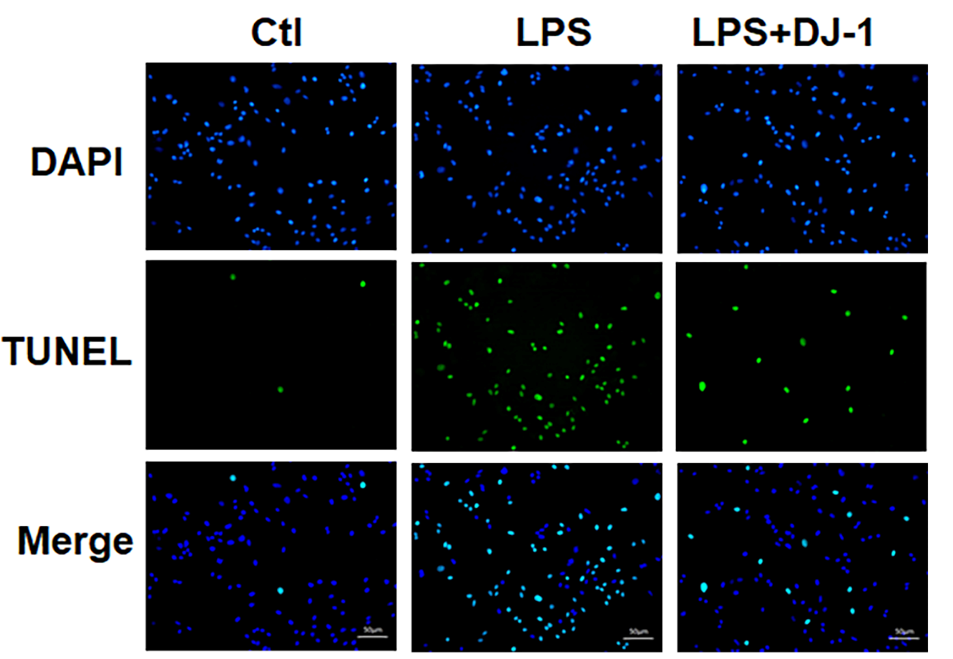
**Supplementary figure 3.** L2 cells were transfected with DJ-1 or NC and treated with LPS. Apoptosis cell numbers were tested by TUNLE staining.


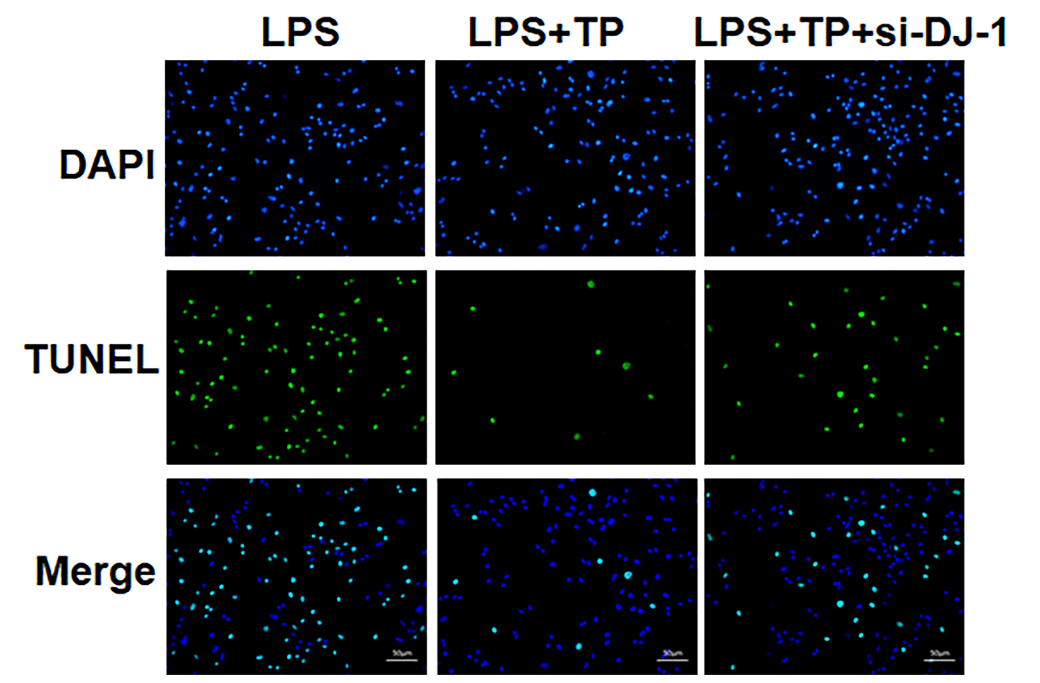
**Supplementary figure 4.** L2 cells were treated with LPS with or without TP (15 μg/ml) and transfected with si-DJ-1 or NC. Apoptosis cell numbers were tested by TUNLE staining.
